# Autoclavable Albumin-Based Cryogels with Uncompromising Properties

**DOI:** 10.3390/gels9090712

**Published:** 2023-09-01

**Authors:** Kairui Duan, Nabila Mehwish, Mengdie Xu, Hu Zhu, Jiajun Hu, Mian Lin, Lu Yu, Bae Hoon Lee

**Affiliations:** 1 Postgraduate Training Base Alliance, Wenzhou Medical University, Wenzhou 325011, China; duankairui20210320@163.com; 2 Wenzhou Institute, University of Chinese Academy of Sciences, Wenzhou 325011, China; xumd@wiucas.ac.cn (M.X.); joseph.hu.zhu@gmail.com (H.Z.); hu81333262@126.com (J.H.); linmian@wiucas.ac.cn (M.L.); 3 Department of Optometry, Wenzhou Medical University, Wenzhou 325035, China; 15868202096@163.com

**Keywords:** autoclavable, hydrogel, cryogel, BSAMA, sterilization

## Abstract

The development of autoclavable hydrogels has been driven by the need for materials that can withstand the rigors of sterilization without compromising their properties or functionality. Many conventional hydrogels cannot withstand autoclave treatment owing to the breakdown of their composition or structure under the high-temperature and high-pressure environment of autoclaving. Here, the effect of autoclaving on the physical, mechanical, and biological properties of bovine serum albumin methacryloyl (BSAMA) cryogels at three protein concentrations (3, 5, and 10%) was extensively studied. We found that BSAMA cryogels at three concentrations remained little changed after autoclaving in terms of gross shape, pore structure, and protein secondary structure. Young’s modulus of autoclaved BSAMA cryogels (BSAMA^A^) at low concentrations (3 and 5%) was similar to that of BSAMA cryogels, whereas 10% BSAMA^A^ exhibited a higher Young’s modulus value, compared with 10% BSAMA. Interestingly, BSAMA^A^ cryogels prolonged degradation. Importantly, cell viability, drug release, and hemolytic behaviors were found to be similar among the pre- and post-autoclaved cryogels. Above all, autoclaving proved to be more effective in sterilizing BSAMA cryogels from bacteria contamination than UV and ethanol treatments. Thus, autoclavable BSAMA cryogels with uncompromising properties would be useful for biomedical applications.

## 1. Introduction

A wide range of biomedical applications require hydrogels to be sterilized in order to reduce the risk of infections, which is one of the primary healthcare issues [1]. However, hydrogels are among the most challenging materials to sterilize owing to their sensitivity to heat and radiation as well as the presence of water in their structure. Since one of the mechanisms of biopolymer degradation is hydrolysis, the high water content in hydrogels makes sterilizing procedures difficult [2]. Thus, hydrogels are dried before sterilization for some specific applications. They may then be rehydrated and applied after that [3]. However, for some hydrogel formulations and uses, such as soft contact lenses or bio-inks, drying hydrogels for sterilization is not an option.

Cryopolymerization, or controlled polymerization at extremely low temperatures, has been used recently to create macroporous hydrogels [4,5]. As a result, it is possible to tune the pore size, morphology, and mechanical properties of hydrogels. Cryogels, as an example, differ from conventional hydrogels in that they display a 3D network of massive interconnected pores, highly crosslinked polymer walls, and excellent mechanical capabilities [6]. Cryogels can also be made for suturable intraoral grafts [7], and in vitro models that may replicate the microenvironments of the liver [8]. More importantly, cryogels support a somewhat strong resilience to autoclave (moist heat) sterilization, which is used as one of the terminal sterilization methods [4].

The benefits of employing moist heat as a sterilization technique are its effectiveness, rapidity, simplicity, low cost, and lack of harmful waste production [9,10]. With varying degrees of success, a number of groups have investigated how autoclaving affects the characteristics of hydrogels and cryogels. For instance, Takei et al., investigated how autoclave treatment affected cryogels made of physically crosslinked chitosan-gluconic acid for use as wound dressings [11]. They demonstrated that the thermal resistance of cryogels was increased by the ratio optimization of chitosan to gluconic acid. However, the physical characteristics of autoclaved cryogels significantly deteriorated, and the gels shrank by around 40%. Similarly, Pierre Villard et al., examined the properties of a variety of cryogels (including hyaluronic acid glycidyl methacrylate (HAGM), methacrylated alginate (MA-Alginate), and methacrylated gelatin (MA-Gelatin)), which were little affected by autoclave sterilization [4,12], in contrast to their respective hydrogels. The physical properties of HAGM and MA-Alginate cryogels did not change after autoclave treatment but their cell-adhesive properties were not good enough compared to MA-Gelatin cryogels. On the other hand, the Young’s modulus of MA-Gelatin cryogels significantly decreased from 17 to 9 kPa after autoclaving. Additionally, the swelling ratio of autoclaved MA-Gelatin cryogels decreased by 6%. Still, avoiding some of the damages to hydrogels and cryogels during the autoclave sterilization remains challenging.

In this work, inspired by the improved functionalities of albumin gels [13], we have prepared bovine serum albumin methacrylate (BSAMA) cryogels and compared albumin cryogel properties before (BSAMA cryogels) and after autoclaving (BSAMA^A^ cryogels), in terms of the swelling ratio, pore connectivity, mechanical strength, degradation, and drug delivery. Additionally, the cell viability, proliferation, and hemolytic behavior of BSAMA and BSAMA^A^ cryogels were also evaluated. To the best of our knowledge, this is the first report on the autoclavable albumin-based cryogels.

## 2. Results and Discussion

### Characterization of BSAMA and BSAMA^A^ Cryogels

Cryogelation generally produces macroporous scaffolds [7]. Cryogels show large porous structures formed by melting ice crystals after cryogelation at subzero temperatures [14]. They have adjustable mechanical strength, suitable pore structures, and good biocompatibility. Pore size is a very important evaluation index of cryogels, and pore size closely influences cell adhesion, migration, and proliferation. The large pore structure of the scaffold is conducive to cell migration, facilitating the transport of nutrients, water, and gas and the removal of metabolic wastes [15]. In addition, the optimal pore size of scaffolds varies with different cell types; for example, fibroblasts bind well to the pores of 40–150 μm, the optimal pore size for liver tissue regeneration is 45–145 μm, and the pore size for bone regeneration is 100–400 μm [16]. The pore size can be changed by adjusting the concentrations of the crosslinking compound and the crosslinking agent [7].

**Figure 1:**
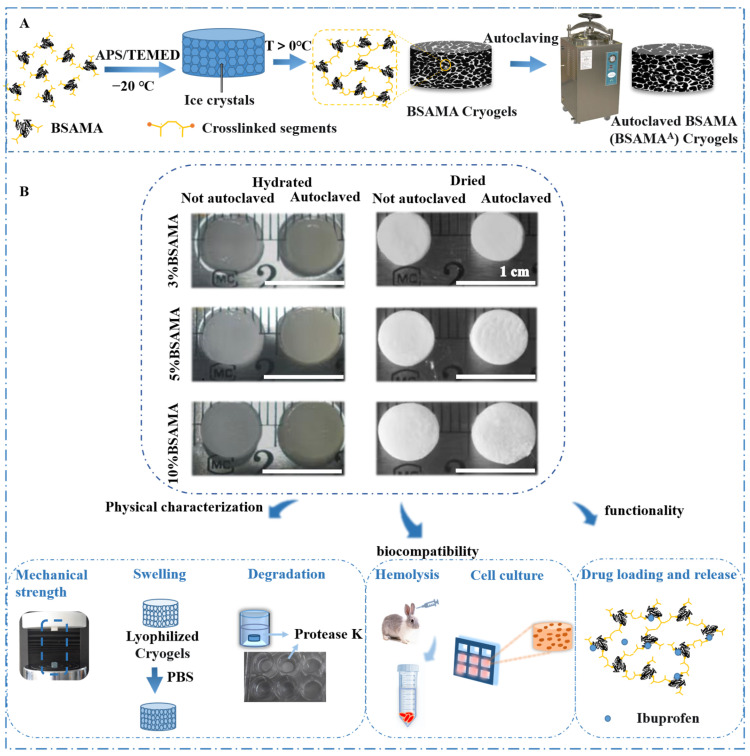
Design of robust BSAMA cryogel scaffolds sustaining autoclave sterilization. (**A**) Schematic depicting the process of BSAMA cryogel fabrication and autoclaving. (**B**) Shape fidelity testing of non-autoclaved and autoclaved BSAMA cryogels in the wet and dry state. Photographs showing shape fidelity of cryogels made from three different polymer concentrations (BSAMA: 3, 5, and 10% *w*/*v*) before and after autoclaving. As-prepared cryogels were further tested for the effect of autoclave sterilization on the physical, biocompatible, and other functional characteristics. The scale bar in the figure represents 1 cm.

The gelation process of BSAMA cryogels was carried out at a low temperature (−20 °C), with a catalyst (N,N,N′,N′-tetramethylethylenediamine: TEMED) and a free radical initiator (ammonium persulfate: APS), to crosslink BSAMA to form cryogels. BSAMA cryogels with different concentrations (3, 5, and 10%) were prepared by a simple strategy of cryogelation (Figure 1A). BSAMA cryogels (before autoclaving) and BSAMA^A^ cryogels (after autoclaving) with 3%, 5%, and 10% showed little difference in shape and size. BSAMA^A^ cryogels appeared slightly yellow (Figure 1B). In addition, the effect of autoclave sterilization on the cryogel properties, including physical, biocompatible, and other functional characteristics, was further examined.

**Figure 2:**
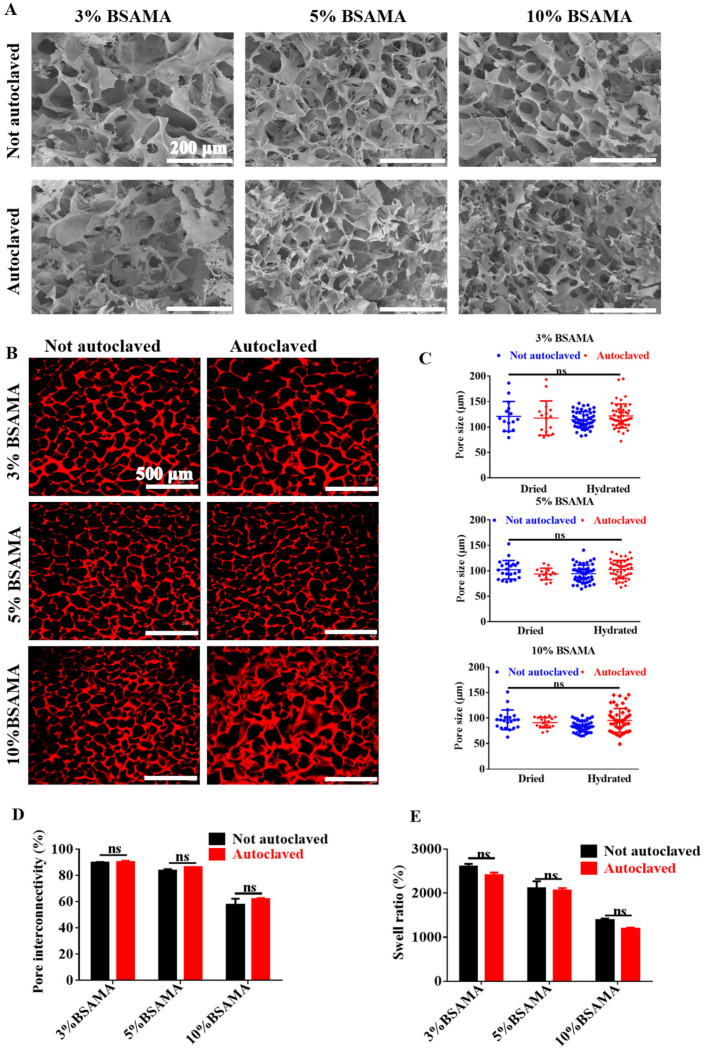
Structural characterization of BSAMA scaffolds in both wet and dry conditions. (**A**) Scanning electron microscopy (SEM) images and (**B**) confocal laser scanning microscopy (CLSM) images of BSAMA cryogels before and after autoclaving. (**C**) Analysis of pore size distribution, calculated using ImageJ (version 1.8.0). (**D**) Pore interconnectivity presented as a porosity ratio and (**E**) swelling ratio presented as water content plotted against the protein concentration. ns denotes ‘not significant’ (*n* = 3).

First, the autoclaved (BSAMA^A^) and not autoclaved (BSAMA) cryogels were evaluated in terms of pore structure using SEM. Figure 2A displays the representative SEM images of BSAMA and BSAMA^A^ cryogels in the dry state. In accordance with previous reports [6,8], the concentration of BSAMA was inversely proportional to the pore size: the pore size of 3% BSAMA and BSAMA^A^ cryogels was about 80–130 μm, and the pore size range of cryogels at 5% and 10% further decreased to 71–109 μm and 58–85 μm, respectively. Both BSAMA and BSAMA^A^ cryogels displayed a similar macroporous structure with thick walls. This is further supported by laser scanning confocal microscopic images (Figure 2B) of the cryogels in the wet state, where the macropores of BSAMA^A^ cryogels remained intact after autoclaving. These results are consistent with other kinds of cryogels, such as HAMA, MA-Alginate, and MA-Gelatin [17]. The cryogels are known to have better resistance to high temperature and high pressure than the traditional hydrogels, maintaining their shape and pore structures during autoclaving. Interestingly, 10% BSAMA^A^ cryogels were observed to have significant pore wall thickening, as compared to 10% BSAMA cryogels. Moreover, the pore size in the dry state appeared slightly larger than that in the wet state for both BSAMA and BSAMA^A^ cryogels at different concentrations, as shown in Figure 2C. In addition, as seen in Figure 2D, the higher the concentration of BSAMA and BSAMA^A^ cryogels, the smaller the pore interconnectivity (PI). The pore size distribution for wet cryogels fell roughly in the same range regardless of the autoclave treatment, but the average pore size tended to be slightly larger for autoclaved samples (BSAMA^A^ cryogels). Figure 2D shows that the PI of BSAMA and BSAMA^A^ cryogels at 3 and 5% was relatively high (i.e., >75%), indicating that the pores were well connected in the cryogels. Interestingly, the PI of 3, 5, and 10% BSAMA^A^ cryogels increased slightly as compared to its BSAMA counterparts, but there was no statistical difference. Additionally, the swelling ratio of BSAMA and BSAMA^A^ cryogels was examined, which can be used to measure the water absorption capacity of cryogels [18,19]. The swelling ratios of BSAMA and BSAMA^A^ cryogels reached their equilibrium within 45 min (Figure S2). As seen in Figure 2E, they were dependent on their concentrations. The higher the concentration of BSAMA and BSAMAA cryogels, the smaller the swelling ratio. BSAMA^A^ cryogels swelled slightly less than BSAMA cryogels, especially at 3% and 10%, which was not significantly different.

At the same time, as a scaffold, hydrogel stiffness is also an important parameter. When cryogels are used as a scaffold for tissue engineering, cells need to match their mechanical properties to facilitate better cell proliferation and migration, such as a human healthy liver model (6 kPa) and a cirrhosis model (29 kPa) [8]. Steam sterilization may possibly cause the loss of mechanical properties of hydrogels [1]. A previous study reported that the Young’s modulus of GelMA cryogels was reduced after autoclaving, whereas the Young’s modulus of HAGMA cryogels was little affected [4,12]. 

**Figure 3:**
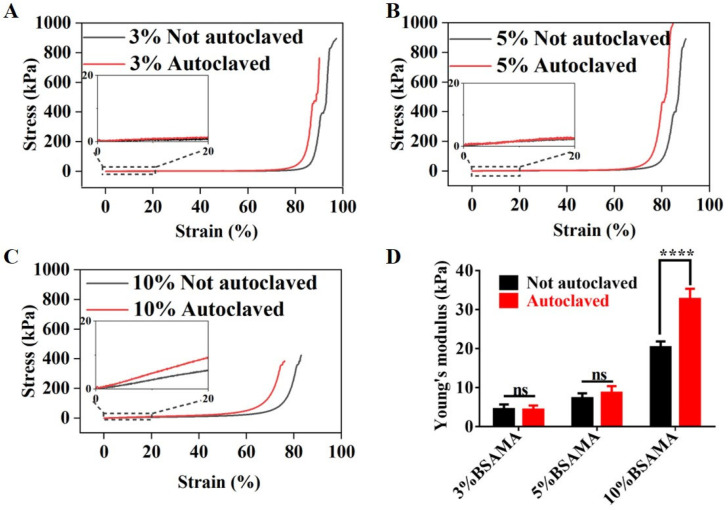
Physical characterization of BSAMA and BSAMA^A^ cryogels. Stress–strain curves of (**A**) 3%, (**B**) 5%, and (**C**) 10% BSAMA cryogels before and after autoclave sterilization. (**D**) Young’s modulus, as determined by the slope of the stress–strain curve from 0 to 20% strain (*n* = 3), plotted against the protein concentration. **** *p* < 0.0001; ns: not significant (*n* = 3).

The mechanical strength of BSAMA and BSAMA^A^ cryogels was measured and Young’s modulus was determined by the slope of the stress–strain curve, from 0% to 20% strain. Figure 3 presents the stress-strain curves of 3% (Figure 3A), 5% (Figure 3B), and 10% (Figure 3C) BSAMA and BSAMA^A^ cryogels, with the Young’s modulus reflecting the stiffness of cryogels, presented in Figure 3D. There is a direct correlation between the Young’s modulus and the concentration of BSAMA and BSAMA^A^ cryogels. For BSAMA cryogels at a low concentration, the Young’s modulus was little influenced by autoclaving: BSAMA cryogels with 3% and 5% exhibited a Young’s modulus of nearly 3–5 kPa and 5–8 kPa, respectively. Following autoclave sterilization, the Young’s modulus of 3% and 5% BSAMA^A^ cryogels stayed almost unchanged, with values at 3–5 kPa and 7–10 kPa, respectively. However, the Young’s modulus of 10% cryogels significantly increased after autoclave sterilization, from 19–22 kPa (10% BSAMA) to 29–35 kPa (10% BSAMA^A^). BSAMA^A^ cryogels seemed more brittle at large strains than BSAMA cryogels. Judging from the fact that the pore walls of BSAMA^A^ cryogels were observed to be thickened in Figure 2B, the physical interactions of BSAMA^A^ cryogels were presumed to be further enhanced by autoclaving [11]. Thus, the increase in stiffness and brittleness might result from partial physical crosslinking within BSAMA chains under high temperature and high pressure.

**Figure 4:**
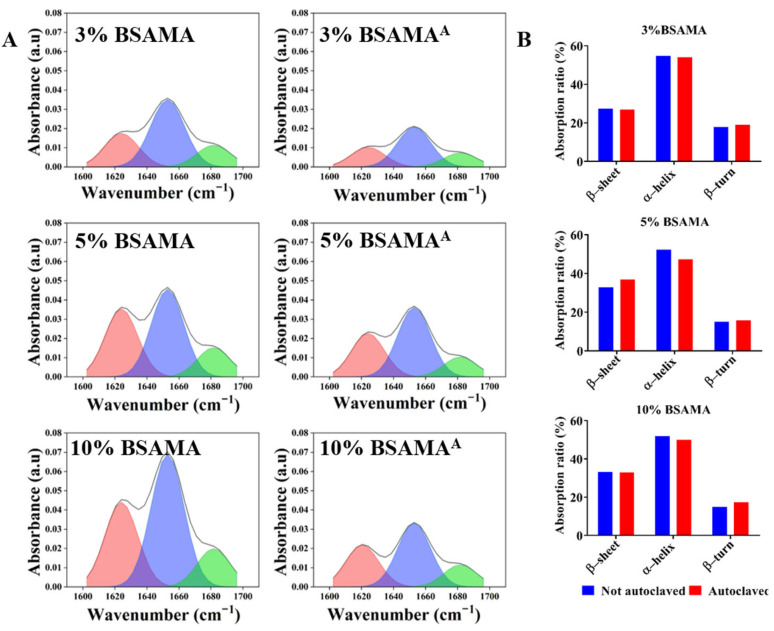
Secondary structure analysis by FTIR for both BSAMA and BSAMA^A^. (**A**) Absorption map of secondary structures of the protein at the amide I band. Red: β-sheet, blue: α-helix, and green: β-turn. (**B**) Absorption proportion of secondary structures (α-helix, β-sheet, and β-turn).

FTIR represents an easy tool to monitor the secondary structure of BSAMA proteins before and after autoclaving (Figure 4). The infrared absorption peak of BSAMA cryogels was measured after they were freeze-dried and then ground into powder. In general, the secondary structure (α-helix, β-sheet, and β-turn) changes of BSAMA cryogels before and after autoclaving appeared marginal. The α-helix contents of 3%, 5%, and 10% BSAMA cryogels were nearly 54.85%, 52.20%, and 51.91%, respectively. Following autoclave sterilization, the α-helix contents of 3%, 5%, and 10% BSAMA^A^ cryogels were 54.08%, 47.34%, and 47.85%, respectively. As can be seen in Figure S1, no new covalent bonds seemed to be formed in BSAMA^A^ cryogels after autoclaving. In addition, an overall reduction of absorbance of the autoclaved BSAMA^A^ cryogels was observed, which might be due to the steric effect of more densely packed autoclaved BSAMA^A^ cryogels, as compared to BSAMA cryogels.

**Figure 5:**
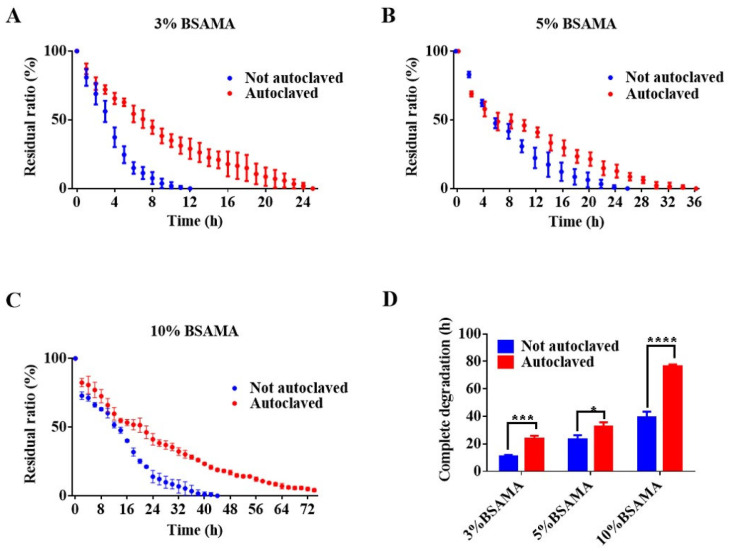
Enzymatic degradation of BSAMA and BSAMA^A^ cryogels in 0.1 mg mL^−1^ of Proteinase K in PBS (pH 7.4) at 37 °C. Degradation curves of (**A**) 3%, (**B**) 5%, and (**C**) 10% BSAMA and BSAMA^A^ cryogels. (**D**) Comparison of complete degradation time of 3%, 5%, and 10% BSAMA and BSAMA^A^ cryogels. * *p* < 0.05, *** *p* < 0.001, and **** *p* < 0.0001 (*n* = 3).

The degradation property of hydrogels is another important indicator for tissue engineering. Cryogels can support the sustained release of drugs under enzyme degradation for cell culture [20]. As a drug-carrying biomaterial injected into the body, hydrogels with appropriate biodegradability are easily metabolized, which is a very important safety indicator. For BSAMA, most of the metabolites are amino acids, which are natural compounds and may exhibit good biocompatibility. The in vitro enzymatic degradation of BSAMA and BSAMA^A^ cryogels was tested using a protease enzyme (Proteinase K). Proteinase K is a broad-spectrum protease, which has good hydrolytic activity to natural proteins under mild and neutral conditions [21]. After the initial mass, *m*0, of the swelling cryogel was measured, the BSAMA cryogel was put into a 1 mL Proteinase K solution (0.1 mg mL^−1^), Proteinase K was replaced every 2 h, and the mass of the cryogel was measured every 1 h to determine the degradation rate. It was found that the degradation rate was dependent on the concentration of the polymer, with prolonged degradation observed at a higher concentration. The complete degradation time was found to be 10–12 h, 20–26 h, and 36–44 h for BSAMA cryogels at 3%, 5%, and 10%, respectively (Figure 5). Following autoclave sterilization, the complete degradation time for BSAMA^A^ cryogels increased to 21–25 h, 30–36 h, and 76–78 h, respectively. Autoclaving has been reported to possibly accelerate the degradation of hydrogels [1]. Interestingly, the degradation rate of BSAMA^A^ cryogels (after autoclaving) appeared slower than that of BSAMA cryogels. 

**Figure 6:**
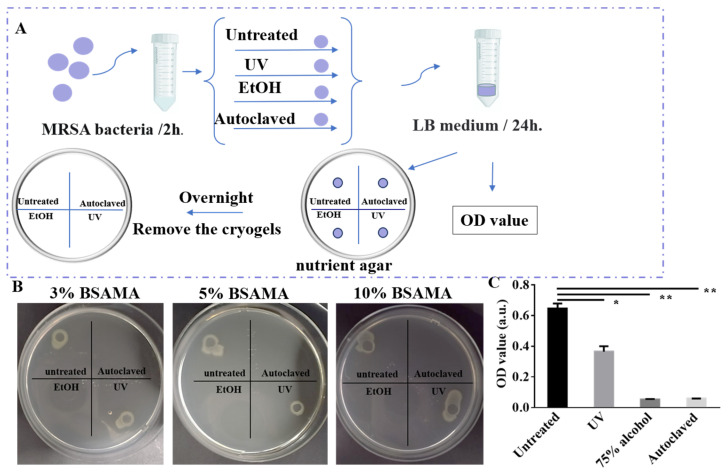
Therapeutic effects of three sterilization methods on BSAMA cryogels. (**A**) The operation process of different sterilization methods. (**B**) Bacterial growth zones around MRSA-contaminated BSAMA cryogels with or without autoclaving, ethanol, and UV irradiation sterilization. (**C**) OD values were measured to quantify bacterial growth on 5% BSAMA cryogels. * *p* < 0.05, ** *p* < 0.01 (*n* = 3).

The therapeutic effect of autoclaving on bacteria adhesion/growth was then investigated in parallel with two other common sterilization methods, namely 75% ethanol and UV light (Figure 6). As shown in Figure 6A, the cryogel soaked in bacteria for two hours was disinfected by four treatment methods (autoclave, 75% alcohol, ultraviolet disinfection, and no treatment), then cultured in a LB solution for 24 h, the OD value of the extracted solution was measured, and the cryogel was cultured in the nutrient agar overnight. There was no bacterial growth around BSAMA cryogels sterilized by autoclaving and 75% ethanol (Figure 6B), indicating that these two sterilization methods are effective (*p* < 0.01, *n* = 3). Figure 6C exhibits the growth number of bacteria stored in LB medium overnight after sterilization. UV had a certain sterilization effect, but there was still bacterial growth similar to the untreated control group, while autoclaving and 75% alcohol exerted a better sterilization effect. Such an observation is consistent with a previous report [4]. Although ethanol treatment disinfected the gels, their SEM results showed that there were still some bacterial residues on the cryogel surface. Thus, it can be concluded that only autoclave treatment ensures comprehensive sterilization without any bacterial debris or residues.

As an important carrier protein in the body, albumin can be combined with many small molecules of organic substances and inorganic ions in the body to form easily soluble complexes in the plasma and to facilitate the transportation and metabolism of these insoluble drugs [22]. Therefore, the internal hydrophobic and external hydrophilic structure of BSA can well encapsulate and become an effective carrier of hydrophobic drugs [23,24]. Importantly, the secondary structure of albumin can be involved in ligand-binding properties. After autoclave sterilization, the FTIR spectra showed no new bond formation, and the protein secondary structure (α-helix) remained little changed, with no statistical significance. Therefore, the secondary structure of BSAMA cryogels was little affected by steam sterilization.

**Figure 7:**
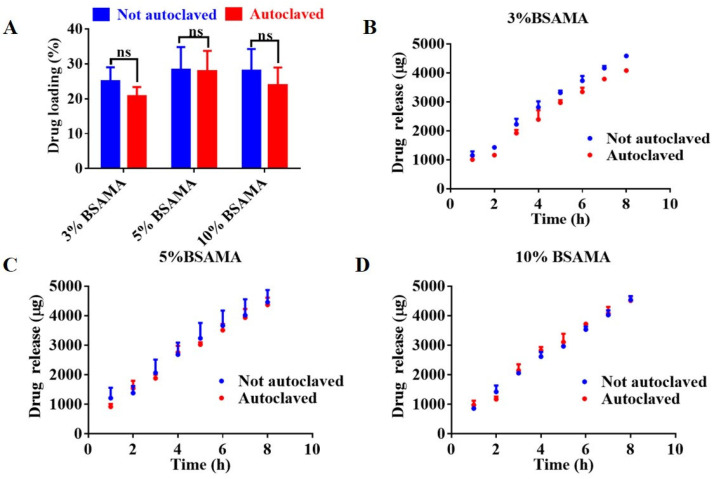
Loading and release profile of a hydrophobic drug (ibuprofen) by 3%, 5%, and 10% BSAMA before and after autoclave sterilization. (**A**) Drug loading of 3%, 5%, and 10% BSAMA before and after autoclave sterilization. The loading was achieved by soaking in a 22 mg mL^−1^ ibuprofen solution for 24 h. (**B**–**D**) The cumulative ibuprofen release was measured as a function of time for 8 h from 3%, 5%, and 10% BSAMA cryogels, respectively, before and after autoclave sterilization. ns: not significant (*n* = 3).

The influence of autoclaving on the drug loading and drug release profile of BSAMA cryogels was also investigated. Ibuprofen was loaded into the cryogel by an immersion method. After the cryogel was immersed in a high concentration of ibuprofen for 24 h, the concentration of ibuprofen in the solution was measured with an ultraviolet spectrophotometer to obtain the amount of ibuprofen loaded into the cryogel. After that, the cryogel was put into PBS to measure ibuprofen release at designated time points (in accordance with the standard curve (Figure S3)). As seen in Figure 7A, both BSAMA and BSAMA^A^ cryogels exhibited good drug loading efficiency (>20%): the drug loading of BSAMA^A^ cryogels at each concentration was slightly smaller than that of BSAMA cryogels, though there was no statistical difference.

Ibuprofen is a hydrophobic drug that interacts with BSA or BSAMA via hydrophobic interactions. Figure S4 also shows a slight increase in the surface negative charge of BSAMA^A^ (after autoclaving), which might marginally affect ibuprofen binding. Solute diffusion and scaffold degradation were reported to be the main driving forces for drug release [25]. BSAMA and BSAMA^A^ cryogels exhibited a similar release profile (Figure 7C,D). The drug release amount of BSAMA^A^ cryogels (3%) after sterilization was slightly lower than that of pristine BSAMA cryogels. This may be because after autoclaving, the degradation rate of BSAMA^A^ cryogel was slowed down, resulting in a slower drug release. Nevertheless, BSAMA^A^ cryogel (after autoclaving) still presented comparable drug loading and release characteristics.

**Figure 8:**
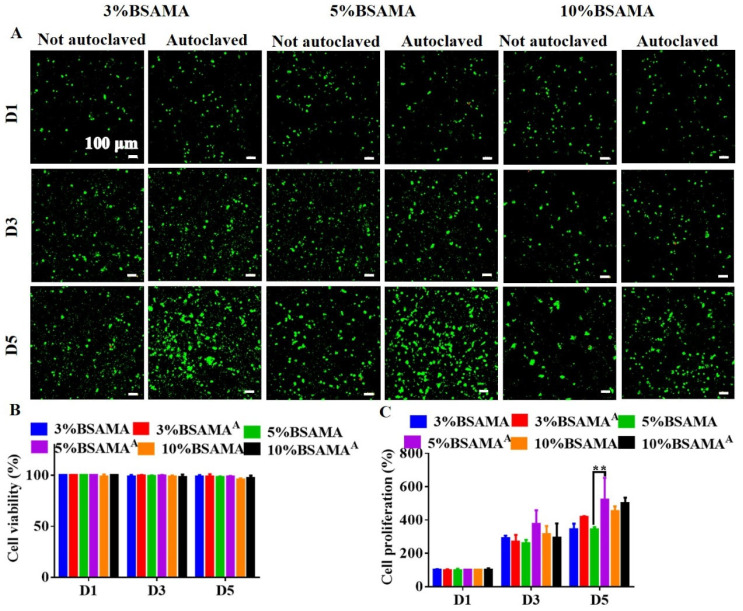
In vitro cell biocompatibility evaluation of cryogels before (BSAMA) and after autoclaving (BSAMA^A^). (**A**,**B**) Cell viability was assessed using live/dead staining under confocal laser scanning microscopy (CLSM): live cells were stained green using calcein AM while dead cells were stained red using EthD-1. (**C**) The cell proliferation of HepG2 cells treated by BSAMA and BSAMA^A^ cryogels with different concentrations for 1, 3, and 5 days (D1, D3, and D5), respectively. ** *p* < 0.01 (*n* = 3).

The in vitro cell culture behavior of the biomaterials can envision their biocompatible attributes for biomedical applications. A porous scaffold with appropriate pore interconnectivity provides the microenvironment for cell adhesion, proliferation, migration, and infiltration [26]. Herein, two types of cells (HepG2 (Figure 8) and L929 (Figure S5)) were used in two separate cell culture experiments to demonstrate the biocompatibility of these autoclavable cryogels. Live/dead and CCK-8 assays were used to evaluate the cell viability and proliferation in BSAMA and BSAMA^A^ cryogels. The cell culture experiments were carried out under aseptic conditions, and the solutions were filtered aseptically to avoid the influence of bacteria on cells. As seen from Figure 8, most of the HepG2 cells in BSAMA and BSAMA^A^ cryogels were alive during the cell culture testing period and grew over time: the cell viability of BSAMA and BSAMA^A^ cryogels was above 95%. CCK-8 results demonstrated that both BSAMA and BSAMA^A^ cryogels supported cell proliferation during the culture period of 5 days. Interestingly, the proliferation of BSAMA^A^ cryogels at day 5 was higher than that of BSAMA cryogels; especially, 5% BSAMA^A^ cryogels more significantly supported cell proliferation than 5% BSAMA cryogels. In addition, L929 cells on 3% and 5% BSAMA^A^ cryogels showed significant proliferation (Figure S5). It is postulated that autoclaving may render the microenvironments (including pore structures and mechanical properties) of BSAMA^A^ cryogels still suitable for 3D cell culture. 

Hemolysis is likely to occur when medical equipment is used in clinical practice. Hemolysis will cause a significant reduction of red blood cells, weakened oxygen transfer ability, and tissue damage. At the same time, after the occurrence of hemolysis, the body will have an emergency response and form thrombosis, which is liable to cause tissue necrosis [27,28], and hemolysis may cause adverse effects on the health of patients [29]. Hemolysis is a process in which the contents of red blood cells (such as hemoglobin) are released from the destruction of red blood cells. The hemolysis ratio is usually divided into the following types: the 0–2% hemolysis ratio is non-hemolytic, the 2–5% hemolysis ratio is low hemolytic, and the 5% hemolysis ratio is highly hemolytic [30,31,32]. Therefore, it is also very important to test the blood compatibility of BSAMA and BSAMA^A^ cryogels.

**Figure 9:**
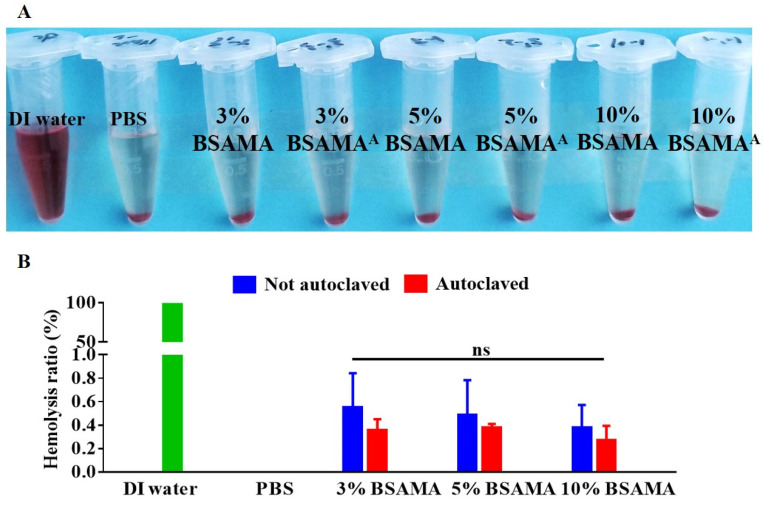
Hemocompatibility of cryogels before (BSAMA) and after autoclaving (BSAMA^A^). (**A**) Photographs of BSAMA and BSAMA^A^ cryogels and control samples after the hemolysis testing. (**B**) Hemolysis ratio (%) of the negative control group (PBS)/positive control group (DI water) and the BSAMA/BSAMA^A^ cryogels at 3, 5, and 10%. ns: not significant (*n* = 3).

Rabbit blood was collected and centrifuged to extract red blood cells. Each cryogel, the negative control (PBS), and the positive control (DI water) were separately added to a solution of obtained red blood cells, and then the hemolysis ratio was calculated. Figure 9A,B show the hemolysis results of BSAMA and BSAMA^A^ cryogels and control groups (DI water and PBS). The positive control group (DI water) appeared to be red, indicating a complete hemolysis of red blood cells in DI water. In contrast, in the negative control group (PBS), red blood cells sunk to the bottom, and there was no rupture of red blood cells. In BSAMA and BSAMA^A^ cryogels, red blood cells were visible at the bottom of the tubes, and the supernatant appeared clear, similar to that of red blood cells in PBS. As presented in Figure 9B, all cryogels exhibited a hemolytic ratio lower than 1% (0–2% hemolysis ratio is non-hemolytic). Interestingly, the hemolytic ratios of 3%, 5%, and 10% BSAMA^A^ cryogels showed slightly lower values than those of 3%, 5%, and 10% BSAMA cryogels, indicating that BSAMA and BSAMA^A^ cryogels had no hemolysis properties and had good blood compatibility, and autoclaving BSAMA cryogels may further improve their biocompatibility. However, no significant difference was observed.

Overall, autoclaving exerted little detrimental influence on the physical properties (pore size, rigidity, and swelling), cytocompatibility, and blood compatibility of BSAMA cryogels.

## 3. Conclusions

In this study, the physical, mechanical, and biological characteristics of BSAMA cryogels (before autoclaving) and BSAMA^A^ cryogels (after autoclaving) were examined. At different concentrations (3, 5, and 10%), BSAMA cryogels showed little detrimental effect of autoclaving on their physiological properties. BSAMA cryogels and BSAMA^A^ cryogels did not significantly vary in drug release or hemolytic properties. Interestingly, autoclaved BSAMA^A^ cryogels at 10% exhibited better mechanical characteristics, and slower degradation. Most notably, compared to untreated, UV, and ethanol treatments, autoclaving of BSA cryogels proved the superior sterilizing approach in terms of no bacterial growth. The autoclavable BSAMA cryogels have the potential to resist the rigorous sterilization process with their features or functions little compromised. These autoclavable albumin cryogels could be applied in a variety of sectors, including biotechnology and medical equipment.

## 4. Materials and Methods

### 4.1. Materials

BSAMA, with a degree of substitution of 100%, was obtained from Wenzhou Shuhe Biotechnology Co., Ltd. (Wenzhou, China). Ammonium persulfate (APS, >98%) and N,N,N′,N′-tetramethylethylenediamine (TEMED, ≥99.5%) were obtained from Rhawn of Shanghai Yi En Chemical Technology Co., Ltd. (Shanghai, China). Proteinase K and anhydrous alcohol were purchased from Aladdin Chemistry Co., Ltd. (Shanghai, China). Dulbecco’s modified Eagle’s medium (DMEM), fetal bovine serum (FBS), penicillin/streptomycin (P/S), live/dead staining (EthD-1/calcein AM), and Rhodamine were supplied by Thermo Fisher Scientific Inc. (Waltham, MA, USA). The Cell Counting Kit-8 (CCK-8) was purchased from Dojindo Molecular Technologies (Dojindo, Japan). Ibuprofen was purchased from Aladdin Chemistry Co., Ltd. (Shanghai, China). Disposable vacuum blood collection containing sodium citrate was obtained from Shijiazhuang Kangwei Medical Equipment Co., Ltd. (Shijiazhuang, China). Nutrient agar was purchased from Wenzhou Kangtai Biotechnology Co., Ltd. (Wenzhou, China). All the reagents were used as received.

### 4.2. Fabrication of BSAMA Cryogels

Bovine serum albumin methacryloyl (BSAMA) cryogels were prepared according to a previous report [8]. Briefly, BSAMA powder was dissolved in PBS to form a clear BSAMA solution of 3%, 5%, and 10%. Each BSAMA solution was mixed with 0.6% ammonium persulfate (APS) as an initiator and 0.25% tetramethylethylenediamine (TEMED) as a catalyst in an ice bath. Resulting solutions were quickly transferred to pre-frozen cylindered-shaped silicone molds [7] to obtain a certain shape of cryogels, and then quickly stored at −20 °C for 24 h to form cryogels. After that, the formed cryogels were thawed at room temperature for 30 min, taken out, and washed three times with a PBS solution. After swelling in the PBS solution for 24 h, the cryogels were stored at 4 °C until future use.

### 4.3. Sterilization of BSAMA Cryogels

As-prepared BSAMA cryogels with three different polymer concentrations (BSAMA: 3, 5, and 10% *w*/*v*) were put into autoclavable plastic bottles and sterilized at 121 °C for 20 min in an autoclave (BL-50A, Shanghai, China), washed three times with PBS, swollen in PBS for 24 h, and eventually stored at 4 °C until future use.

### 4.4. Micro-Structural Imaging of BSAMA Cryogels

Scanning electron microscope (SEM): Microstructural imaging of the dried scaffolds was performed using a transmission FE-SEM (SU8020, Hitachi, Japan) at a voltage of 5 kV. Representative samples were lyophilized for 48 h after being frozen at −80 °C. As-prepared freeze-dried scaffolds were coated with a thin layer of platinum in a sputter to make the surface conductive. The microstructure of BSAMA cryogels was observed under SEM. Multiple images were obtained for each sample, and the pore size was analyzed using ImageJ, with at least 50 aperture analyses per sample.

Confocal laser scanning microscopy (CLSM): The as-prepared BSAMA cryogel scaffolds (3%, 5%, and 10%) in the wet state were observed with a CLSM (A1, Nikon, Japan) before and after autoclaving. After the cryogels were dyed with Rhodamine (1%) for 15 min, each sample was washed with PBS to remove the unbound dye, and the pore size and structure were examined under CLSM. Replicate images of all cryogels were obtained, and their pore sizes were analyzed using ImageJ, with at least 50 pores for each sample.

### 4.5. Physical Characterization of BSAMA Cryogels

Mechanical properties: The mechanical strength of the as-prepared 3%, 5%, and 10% BSAMA cryogels before and after autoclaving was measured using a mechanical testing machine (UTM2102; Shenzhen, China). The compressive stress–strain curves were obtained at 10 mm min^−1^, and Young’s modulus was determined via measuring the slope of the strain–stress curves at strain between 0% and 20%.

Pore interconnectivity and swelling ratio: The swelling behavior of the BSAMA and BSAMA^A^ cryogel scaffolds was observed in PBS for 24 h, with an obtained weight corresponding to *m*1 [4,33]. Cryogel scaffolds were gently placed on Kimwipes to remove the moisture contained in the cryogels and were then weighed as *m*2. After that, cryogels were pre-frozen at −80 °C and freeze-dried for 48 h, obtaining a corresponding weight (*m*0). Lastly, PBS was added into dried cryogels, and after swelling for 24 h, *m*3 was weighed. Three replicate samples of each group were tested (*n* = 3).

$$Swelling\;Ratio\;\left(\%\right)=\frac{\left(m3-m0\right)}{m0}\times 100$$

$$Pore\;Interconnectivity\;\left(\%\right)=\frac{\left(m1-m2\right)}{m1}\times 100$$

### 4.6. Fourier Transform Infrared Spectroscopy (FTIR)

Infrared spectroscopy (tensor II, Brock, Germany) was used to study the binding of covalent amide bonds, and the secondary structure of protein was fitted at the amide I band. The effect of autoclaving on the secondary structure of BSAMA protein was evaluated. Scans were performed in the wavenumber range of 1000–4000 cm^−1^.

### 4.7. Enzymatic Degradation

The as-prepared cryogels were gently wiped using Kimwipes to remove residual water on the surface without removing the internal adsorbed water. The initial weight (*m*0) was weighed, after which a 0.1 mg mL^−1^ Proteinase K solution (pH 7.4) was added to each sample and stored in a shaker at 37 °C with a rotational speed of 150 rpm to measure the mass (*m*1) on the preset time points. The enzyme solution was changed every two hours. Each group had three parallel samples (*n* = 3).

$$Residual\;Ratio\;\left(\%\right)=\frac{m1}{m0}\times 100$$

### 4.8. Analysis of Bacterial Adhesion on BSAMA Cryogels

Drug-resistant MRSA (3.6 × 10^8^ CFU mL^−1^) was added into cryogel scaffolds with three different concentrations (BSAMA: 3, 5, and 10% *w*/*v*) before and after autoclaving and stored in a constant temperature culture shaker (TS-211B, Shanghai, China) at 150 rpm at 37 °C for 2 h. Samples were removed and transferred to centrifuge tubes for sterilization employing different methods (autoclaving, 75% alcohol treatment, UV light irradiation, and no treatment) [4,34]. Autoclaving was performed at 121 °C for 20 min, ethanol treatment was carried out by soaking samples in 75% alcohol for 1 h, and UV treatment was conducted by exposing samples to UV light (254 nm, ≥400 mW cm^−2^) for 1 h. LB medium was added to each treatment for 24 h. OD values were measured by taking out 100 μL of medium. Triplicate samples were tested for each group (*n* = 3).

### 4.9. Drug Loading and Drug Release

Each cryogel was soaked in an ibuprofen solution (22 mg of ibuprofen dissolved in a 1 mL ethanol, *c*0) in a 100 rpm shaker at 37 °C, for 24 h. After taking out each cryogel, the absorbance of a diluted solution (a 0.2 mL sample solution plus 7.8 mL of fresh PBS) at 264 nm was measured with a double-beam ultraviolet visible spectrophotometer (TU-1901, Beijing, China) to calculate drug loading. The ibuprofen concentration (*c*1) of the sample solution was obtained according to a standard curve (Figure S2). Four identical samples of ibuprofen-loaded cryogels were placed in a 48-well plate, a 1.5 mL PBS solution was added into each well, and placed at 37 °C in a constant temperature culture shaker (TS-211B, Shanghai, China) of 100 rpm. After that, a 0.5 mL solution was taken out every hour and a fresh PBS solution of the same volume was added. After diluting the solution taken out every hour, *c*2 was measured with the double-beam ultraviolet visible spectrophotometer (TU-1901, Beijing, China). The cumulative release of the drug was calculated using the standard curve.

$$Drug\;loading\;\left(\%\right)=\frac{\left(c0-c1\times 40\right)}{c0}\times 100$$

Drug Release=c2×Dilution Ratio

### 4.10. In Vitro Biocompatibility of BSAMA Cryogels

#### 4.10.1. Cell Seeding

In this study, immortalized liver hepatocellular carcinoma cells (HepG2 cells) and L929 cells were used as model cells in two separate experiments. HepG2 and L929 cells were obtained from the American Type Culture Collection (ATCC) and cultivated in DMEM with 10% FBS and 1% antibiotic/antimycotic at 37 °C in a 5% CO_2_ atmosphere. The precursor solutions of BSAMA were filtered by a 0.22 μm filter membrane (SCAA-114, Shanghai, China), and the process of cryogels’ preparation was carried out under sterile conditions. All cryogels (before and after autoclaving) were put into a 48-well plate. A generalized term of “cells” was subsequently used for the HepG2 and L929 cells for two separate experiments of cell culture with BSAMA cryogel scaffolds, where 10 μL cell solutions (containing about 20,000 cells) were added to the top of the BSAMA cryogels. Culture media were gently added to each well after 8 h, and the culture media were changed every 2–3 days.

#### 4.10.2. CCK-8

After the cells were loaded into BSAMA and BSAMA^A^ cryogel scaffolds, at specific time points (D1, D3, and D5), cell-laden BSAMA cryogels were transferred to a fresh 48-well plate with a 10% CCK-8 solution and incubated for 4 h at 37 °C in a 5% CO_2_ atmosphere. Then, absorbance was measured.

#### 4.10.3. Live/Dead

The live/dead assay was conducted at specific time points (D1, D3, and D5). After the cells were loaded into BSAMA cryogel scaffolds, cell-inoculated BSAMA cryogels were put into a fresh 48-well plate. DMEM was added with 2 μM of calcein AM (green dye) for the observation of live cells, and DMEM with 4 μM of EthD-1 (red dye) for the observation of dead cells. Samples were stored in an incubator at 37 °C with 5% CO_2_ for 45 min. CLSM was used to observe live/dead cells.

### 4.11. Hemocompatibility of BSAMA Cryogels

Whole blood was collected from the ear border veins of healthy rabbits and placed in reagent tubes containing sodium citrate at a dilution ratio of 1:4 (sodium citrate:blood), which chelated calcium ions in the blood to prevent coagulation reactions. The obtained whole blood was centrifuged for 15 min at a speed of 1000 rpm in a high-speed centrifuge (TGL-16.5M, Shanghai, China) to remove the rich platelet plasma layer in the upper layer and the middle white blood cell layer, and to obtain red blood cells. The obtained red blood cells were washed with PBS 3 times and centrifuged at 2000 rpm for 5 min, and then diluted with PBS to obtain 2% red blood cells (RBC). Cryogels before and after autoclaving were placed in 1.5 mL centrifuge tubes with a 0.5 mL PBS solution and a 0.5 mL RBC (2%) solution and incubated at 37 °C for 1 h. Additionally, 0.5 mL of a 2% RBC solution was added into 0.5 mL of DI water and 0.5 mL of PBS, respectively, as a positive control group and a negative control group. After incubation, centrifugation at 3000 rpm for 5 min was performed, 250 μL of supernatants was added into a 96-well plate, and their OD values were measured at 540 nm using a microplate reader (Bio Tek, Winooski, VT, USA). The formula for calculating the hemolysis ratio is as follows [28,30,35]:

$$Hemolysis\;Ratio\left(\%\right)=\frac{\left(OD_{Sample}-OD_{Negative}\right)}{OD_{Positive}-OD_{Negative}}\times 100$$

where *OD_Sample_* is the absorbance of the sample, *OD_Negative_* is the absorbance in PBS, and *OD_Positive_* is the absorbance in deionized water.

### 4.12. Statistical Analysis

Data were analyzed using MS-Excel, OriginPro 8.5.1, and GraphPad prism 6.02. Unless otherwise noted, triplicate samples were used for each condition, and data were presented as mean ± standard deviation (SD). One-way ANOVA followed by a Bonferroni t-test for multiple comparisons was performed to check for significant differences among groups. Data with *p* < 0.05 were considered statistically significant (*p* < 0.01 was represented as “**” and 0.01 ≤ *p* < 0.05 was represented as “*”).

## Data Availability

The data generated from the study are clearly presented and discussed in the manuscript.

## Supplementary Materials

The following supporting information can be downloaded at: https://www.mdpi.com/article/10.3390/gels9090712/s1, Figure S1: Fourier Transform Infrared Spectrometer (FTIR) of BSAMA cryogels before and after autoclaving; Figure S2: The swelling ratios of BSAMA and BSAMAA cryogels with 3%, 5%, and 10% at varying time points were detected within 480 min; Figure S3: The linear relationship between different concentrations of Ibuprofen and absorbance at 264 nm; Figure S4: The surface charge of a 5% BSAMA solution before and after autoclaving was measured by the zeta potential; Figure S5: The cell proliferation of L929 cells cultured in BSAMA cryogels (before autoclaving) and BSAMA^A^ cryogels (after autoclaving) with 3%, 5% and 10% at day 1, day 3, and day 5 (D1, D3, and D5), respectively.
