# Supplementary material for: Autoclavable Albumin-Based Cryogels with Uncompromising Properties
Source: Gels. 2023 Sep 1;9(9):712. doi: 10.3390/gels9090712 (PMC10530076; doi:10.3390/gels9090712)
Supplement: Supplementary file 1 [file gels-09-00712-s001.zip › gels-2568889-supplementary.pdf]

Autoclavable Albumin-based Cryogels with Uncompromising Properties

Kairui Duan [1,2], Nabila Mehwish [2,*], Mengdie Xu [2], Hu Zhu [2], Jiajun Hu [2], Mian Lin [2], Lu Yu [3], Bae Hoon Lee [1,2,3,*]

1. Postgraduate training base Alliance of Wenzhou Medical University, Wenzhou, 325035, China.

2. Wenzhou Institute, University of Chinese Academy of Sciences, Wenzhou, 325011, China.

3. Department of Optometry, Wenzhou Medical University, Wenzhou, 325035, China.

E-mail: nabilamehwish5@gmail.com; bhlee@wiucas.ac.cn

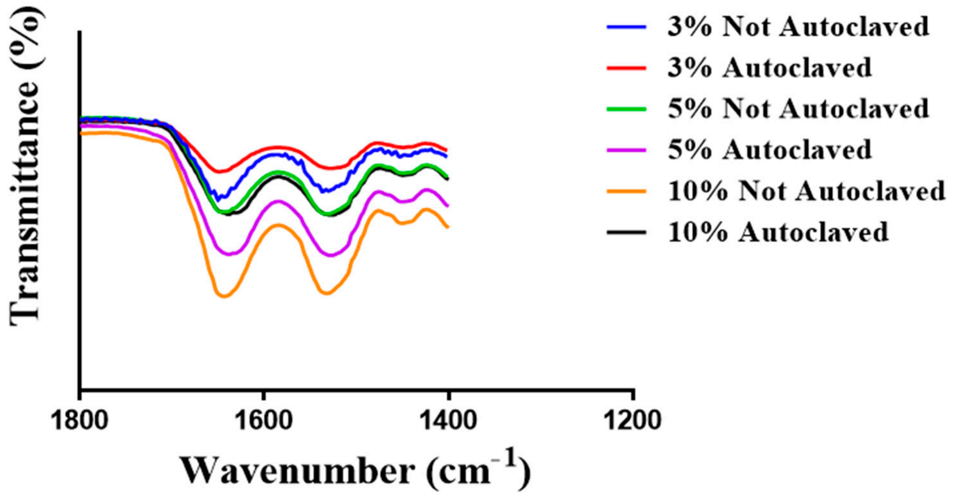

Figure S1. Fourier Transform Infrared Spectrometer (FTIR) of BSAMA cryogels before and after autoclaving.

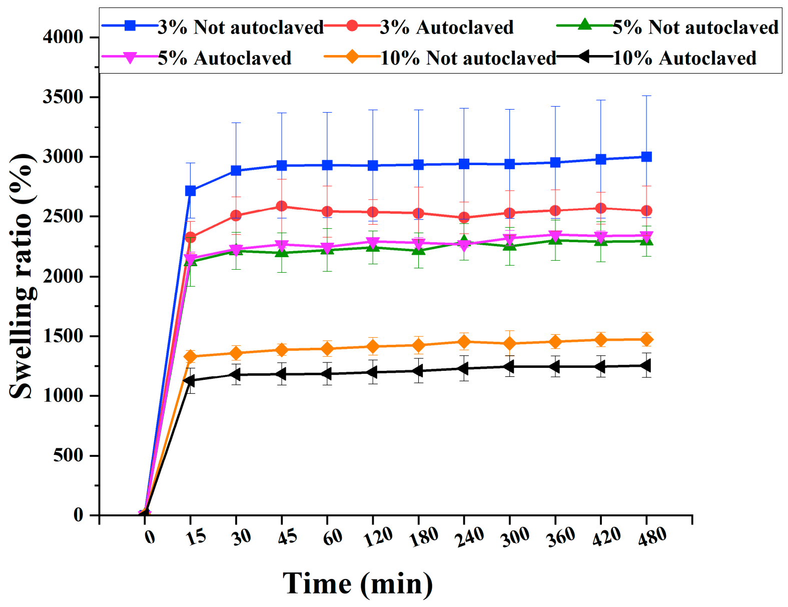

Figure S2. The swelling ratios of BSAMA and $BSAMA^{A}$ cryogels with 3%, 5%, and 10% at varying time points were detected within 480 min.

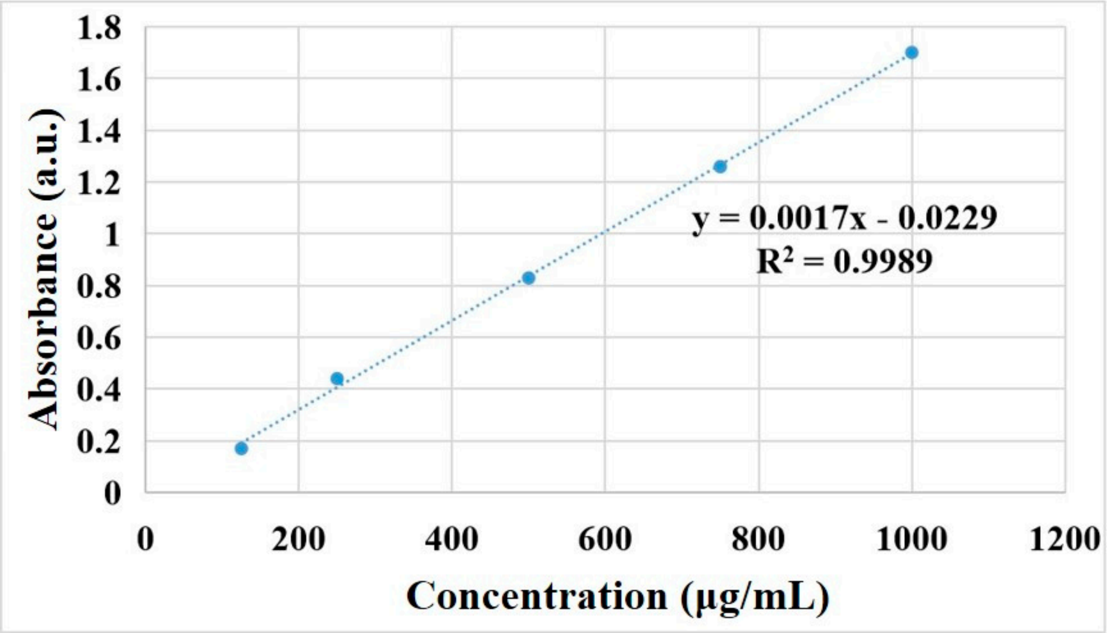

Figure S3. The linear relationship between different concentrations of Ibuprofen and absorbance at 264 nm.

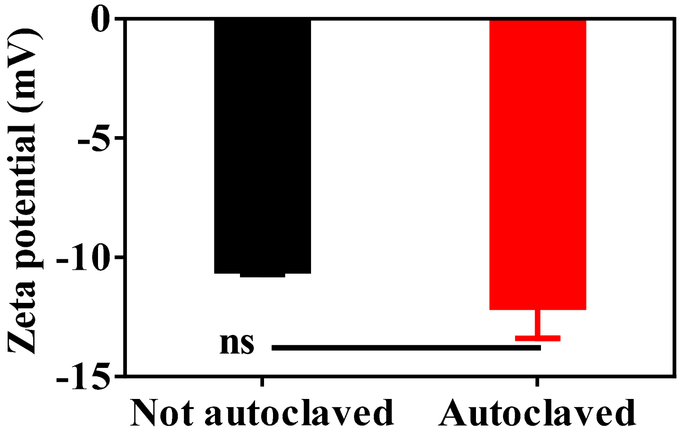

Figure S4. The surface charge of a 5% BSAMA solution before and after autoclaving was measured by the zeta potential.

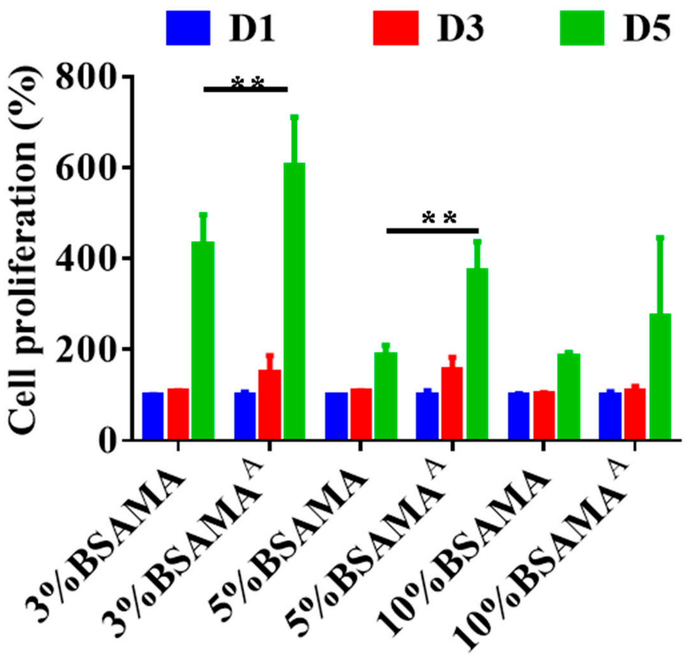

Figure S5. The cell proliferation of L929 cells cultured in BSAMA cryogels (before autoclaving) and BSAMA^A cryogels (after autoclaving) with 3%, 5% and 10% at day 1, day 3, and day 5 (D1, D3, and D5), respectively.
